# Can we read the future from a tree?

**DOI:** 10.7554/eLife.05060

**Published:** 2014-11-14

**Authors:** Michael Lässig, Marta Łuksza

**Affiliations:** 1 **Michael Lässig** is in the Institute for Theoretical Physics, University of Cologne, Cologne, Germanymlaessig@uni-koeln.de; 2 **Marta Łuksza** is in the Institute for Advanced Study, Princeton, United States

**Keywords:** vaccine strain selection, genealogical trees, adaptive evolution, population genetics, viruses

## Abstract

A new method uses genealogies based on sequence data to predict short-term evolutionary patterns.

**Related research article** Neher RA, Russell CA, Shraiman BI. 2014. Predicting evolution from the shape of genealogical trees. *eLife* **3**:e03568. doi: 10.7554/eLife.03568

**Image** In an adapting population, today's individuals descend from high-fitness ancestors

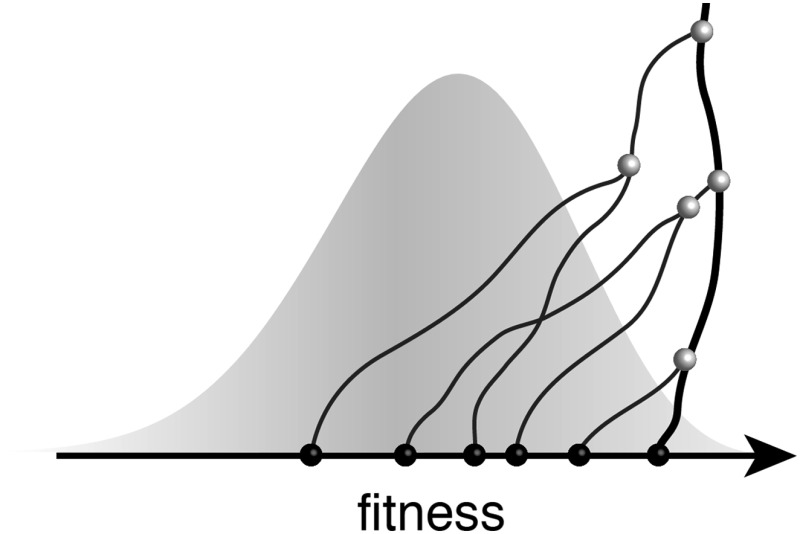

Darwinian evolution is a dynamical principle that connects the past and the future. According to this principle, fitness differences between the individuals in a population are an important driving force of evolution. Biologists have long used fitness effects to explain observed evolutionary changes. For good reasons, however, they have been hesitant to make predictions about the future of a species. Given the bewildering complexity of what is possible in evolution, attempting to say what will happen in a specific instance may appear futile. Moreover, we cannot test any predictions, because we have not seen the evolutionary past and will not see the future.

Recently, however, evolutionary biology is gaining predictive power in an increasing number of systems, which include viruses, bacteria and populations of cancer cells. In these systems, high mutation rates make evolution happen in front of our eyes. Every year, for example, the human influenza virus replaces 2% of the amino acids in the protein domains that interact with the immune system of its host. Using modern genome sequencing, we can now monitor the genetic history of entire populations and reconstruct their genealogical trees. Such trees show how the individuals of today's populations are connected to their evolutionary ancestors. Now, in *eLife*, Richard Neher, Colin Russell and Boris Shraiman investigate how much these trees can tell us about the future of a population (Neher et al., 2014).

Inferring evolutionary patterns from genealogical trees has a long history. Geneticists use probabilistic methods to map mutations onto specific tree branches (Figure 1A). Counting how often these mutations appear in different lineages tells us which fitness effects are predominant in a population (McDonald and Kreitman, 1991; Strelkowa and Lässig, 2012). From the statistics of the genealogical tree itself, epidemiologists infer the growth rate of pathogen populations and use that information to predict the future course of an epidemic (Figure 1B, Stadler, 2010). Neher, Russell and Shraiman—who are at the Max Planck Institute for Developmental Biology, the University of Cambridge, and the University of California at Santa Barbara, respectively—extend this genealogy-based inference to genetic changes within a population (Figure 1C). This required developing new ways to extract information from genealogical trees: predictions must now be made for clades of genetically similar individuals, so we need a model that captures growth rate differences between different clades within one genealogical tree.

**Figure 1.**
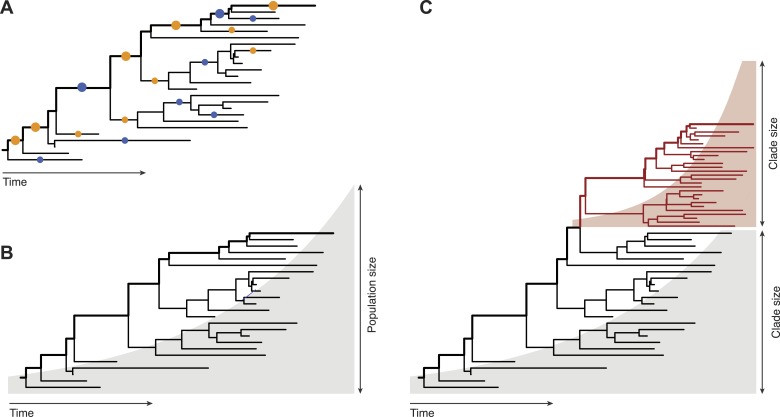
Fitness inference from genealogical trees. Lineages in these trees connect the individuals in a population sample and their evolutionary ancestors, which are the nodes of the tree. Evolutionarily successful lineages have descendants in the far future and are marked by thick lines; all other lineages are lost in the evolutionary process. (**A**) The relative numbers of mutations in successful and in lost lineages measure the predominant fitness effects in a population (orange dots: amino acid changes, blue dots: synonymous mutations). (**B**) The global statistics of nodes and branches measures the absolute rate of exponential population growth (indicated by the shaded area). (**C**) The local statistics of nodes and branches measures growth rate differences between clades. Neher and colleagues use this information to predict clade evolution.

To meet this challenge, Neher and colleagues build on a formalism that is rooted in statistical physics and has become a major new development in population genetics (Tsimring et al., 1996; Rouzine et al., 2003; Desai and Fisher, 2007). The basic idea is simple. Given that fitness differences within a population are carried by genetic mutations, we can imagine splitting each mutation and its fitness effect into ever-smaller pieces. This leads to a model in which the overall fitness variation of a population is made up of many small-effect mutations. By the law of large numbers, the fitness distribution then becomes bell-shaped. Such distributions are called travelling fitness waves (Tsimring et al., 1996). In a given lineage, the accumulation of many small fitness effects follows a diffusive random walk. This picture applies to fast adaptive processes in asexual populations where the expansion of a successful clade is fuelled by multiple beneficial mutations—for example, when viruses evolve to escape their hosts' immune defences.

Neher and colleagues link their fitness wave model to simpler heuristic measures of growth, which can easily be used to analyse data from a large genealogical tree. Specifically, they look at the local tree ‘volume’ *λ*(*τ*), which sums all tree branches in the vicinity of a given node with a discounting scale *τ*. This quantity provides a (nonlinear) measure of how fast the number of individuals grows around that node. For example, in a subtree growing exponentially with rate *r*, the volume *λ*(*τ*) equals simply *τ*/(1–*τr*). By interpreting this growth rate as fitness, Neher and colleagues obtain a measure of fitness differences between clades. A substantial fraction of the local tree volume is generated by small-effect mutations ‘hitch-hiking’ in successful clades (for example synonymous mutations, which do not change a protein). This explains why the local tree volume is closely related to fitness measures used in previous prediction schemes (Łuksza and Lässig, 2014).

The key strength of this method is that it uses only the information contained in a genealogical tree. Thus, it can be applied in cases where we do not know which functions undergo adaptive evolution or where in the genome they are encoded. This feature is also important for interpreting the results: genealogy-based inference reveals growth rate differences within a population sample, but it remains agnostic about their cause. In the fitness wave model, adaptive evolution is that cause, but the demographic structure of a population or variations in sampling density may generate a similar signal in tree data.

Neher and colleagues apply their method to predict the evolution of the human influenza virus A/H3N2. This is a challenging problem: one year in advance, we need to forecast the prevalent clades circulating in a given winter season. Despite the simplicity of their method, Neher and colleagues predict the ancestor sequence of next year's clades with remarkable accuracy for the majority of northern winters between 1995 and 2013.

We do not yet know in detail how the genetic evolution of the influenza virus is related to its interactions with the human immune system. These ‘antigenic’ properties determine how effective influenza vaccines are. They depend on a smaller number of mutations, some of which have individually large effects (Koel et al., 2013). Thus, prediction schemes geared towards antigenic properties must go beyond examining the overall sequence genealogies and weigh mutations by their antigenic effect (Bedford et al., 2014; Łuksza and Lässig, 2014).

Altogether, as Neher and colleagues show, current predictions reach about halfway between random picks and optimal predictions. This poses big conceptual and practical questions: How much can future methods improve on that score? And where does the inherent unpredictability of evolution start? Prediction is the ultimate test of any dynamical principle. Quantitative evolutionary science is being put to that test now.
